# A transmembrane protein family gene signature for overall survival prediction in osteosarcoma

**DOI:** 10.3389/fgene.2022.937300

**Published:** 2022-08-05

**Authors:** Yuehui Du, Xiaohui Zeng, Weiwei Yu, Wei Xie

**Affiliations:** Department of Orthopedics, Pu’ai Hospital, Tongji Medical College, Huazhong University of Science and Technology, Wuhan, China

**Keywords:** transmembrane protein family, osteosarcoma, signature, prognosis, immune microenvironment

## Abstract

The transmembrane (TMEM) protein family is constituted by a large number of proteins that span the lipid bilayer. Dysregulation of TMEM protein genes widely occurs and is associated with clinical outcomes of patients with multiple tumors. Nonetheless, the significance of TMEM genes in the prognosis prediction of patients with osteosarcoma remains largely unclear. Here, we comprehensively analyzed TMEM protein family genes in osteosarcoma using public resources and bioinformatics methods. Prognosis-related TMEM protein family genes were identified by the univariate Cox regression analysis and were utilized to construct a signature based on six TMEM protein family genes (*TMEM120B*, *TMEM147*, *TMEM9B*, *TMEM8A*, *TMEM59*, and *TMEM39B*) in osteosarcoma. The prognostic signature stratified patients into high- and low-risk groups, and validation in the internal and external cohorts confirmed the risk stratification ability of the signature. Functional enrichment analyses of differentially expressed genes between high- and low-risk groups connected immunity with the prognostic signature. Moreover, we found that M2 and M0 macrophages were the most abundant infiltrated immune cell types in the immune microenvironment, and samples of the high-risk group showed a decreased proportion of M2 macrophages. Single-sample gene set enrichment analysis revealed that the scores of neutrophils and Treg were markedly lower in the high-risk group than these in the low-risk group in The Cancer Genome Atlas and GSE16091 cohorts. As for the related immune functions, APC co-inhibition and cytolytic activity exhibited fewer active levels in the high-risk group than that in the low-risk group in both cohorts. Of the six TMEM genes, the expression of *TMEM9B* was lower in the high-risk group than in the low-risk group and was positively associated with the overall survival of osteosarcoma patients. In conclusion, our TMEM protein family gene-based signature is a novel and clinically useful prognostic biomarker for osteosarcoma patients, and *TMEM9B* might be a potential therapeutic target in osteosarcoma.

## Introduction

Osteosarcoma is the most common primary bone cancer that mainly affects individuals at the age of 15–19 years, with a second peak in them at 75–79 years (Rickel et al., 2017). It is a very rare disease that has an incidence of about one to three individuals annually per million (Kansara et al., 2014). Osteosarcoma is a highly malignant cancer and has a propensity for local invasion and early metastasis (Sayles et al., 2019; Sheng et al., 2021). The most common site of distant metastasis is the lung, and lung-only metastases account for approximately 82% of metastatic cases (Gill and Gorlick, 2021). The standard therapy for osteosarcoma patients includes surgery resection and chemotherapy, which has led to a dramatic increase in the overall survival of patients with a localized disease, and the five-year survival rate increased from less than 20 to 70% over the past 30 years (Belayneh et al., 2021; Chen et al., 2021). However, unfortunately, 20–30% of osteosarcoma patients are metastatic or recurrent cases, with the five-year survival rate being less than 20% and remaining stagnant (Saraf et al., 2018). The traditional clinicopathological characteristics including gender, age, and TNM stage are the basis for designing management schedules and prognosis prediction for osteosarcoma patients. However, significant variations in the clinical outcomes of patients have been discovered even for those cases that receive the standard management and harbor similar clinicopathological characteristics (Gill and Gorlick, 2021). Thus, biomarkers that could accurately predict the prognosis in osteosarcoma are urgently needed, which might be beneficial for personalized management and clinical decision.

The transmembrane (TMEM) protein family is constituted by a large number of proteins that span the lipid bilayer and these proteins are components of the biological membranes including the membranes of endoplasmic reticulum, lysosome, mitochondria, and the Golgi apparatus (Beasley et al., 2021; Koteluk et al., 2021). TMEMs are wildly expressed in various types of tissues and are supposed to function as channels to permit the transport of different substances across them (Zhang et al., 2022). Though the function of TMEMs remains largely unknown, emerging evidence reveals the vital roles of TMEMs in tumor occurrence and progression (Schmit and Michiels, 2018; Marx et al., 2020). Recent research studies have reported that the aberrantly expressed TMEM genes in tumors could serve as tumor suppressors or oncogenes and that TMEMs were involved in the regulation of cell proliferation, invasion, metastasis, and chemoresistance (Xu et al., 2019; Zhang et al., 2021). Moreover, several TMEMs were correlated with the overall survival of patients and could act as prognostic biomarkers in multiple tumors. For example, Shiraishi et al. (2021) reported that higher expression of *TMEM180*, a colorectal cancer-specific molecule, predicted worse overall survival in patients with stage III colorectal cancer. A further example includes *TMEM106C*, which functions as an oncogene, and the upregulation of *TMEM106C* was associated with poor prognosis in hepatocellular carcinoma (Duan et al., 2021). A better understanding of TMEM protein family genes thus opens perspectives for the identification of prognostic markers in osteosarcoma.

In the present study, we conducted a comprehensive analysis of TMEM protein family genes in osteosarcoma using public resources and bioinformatics methods. The prognosis-related TMEM protein family genes were identified and utilized for the construction of a prognostic signature in osteosarcoma. The risk stratification ability of the TMEM protein family gene-based signature was validated in both internal and external cohorts. Moreover, we analyzed the association of the prognostic signature with tumor immune cell infiltration and the immune microenvironment.

## Materials and methods

### Data sources

The expression profiles including the RNA-sequencing data and clinical data of patients with osteosarcoma were collected from the cancer genome Atlas (TCGA) database (https://portal.gdc.cancer.gov/). The GSE16091 dataset containing RNA-seq data and corresponding clinicopathological features was downloaded from the Gene Expression Omnibus (GEO) database (https://www.ncbi.nlm.nih.gov/geo/) and was employed for external validation. Patients without complete clinical information were not considered for the study. A total of 86 and 34 osteosarcoma patients were selected from the TCGA and GEO databases for further analysis, respectively.

### Signature construction and validation

In the TCGA osteosarcoma cohort (hereafter referred to as the entire cohort), TMEM protein family genes were subjected to the univariate Cox regression analysis to identify prognosis-related genes using the survival package in R upon the threshold of *P*-value less than 0.05. Then, the entire cohort was randomly separated into a training and a testing cohort at a ratio of approximately 1:1. Then, prognostic TMEM protein family genes were subjected to least absolute shrinkage and selection operator (LASSO) regression analysis to avoid overfitting. Multivariate Cox regression analysis was performed to further screen the candidate genes and calculate the corresponding regression coefficients. A prognostic signature was ultimately constructed based on the linear combination of gene expression levels and regression coefficients. The risk score of each case in the training, testing, entire, and GSE16091 cohorts was calculated using the following formula: $\mathrm{risk}\;\mathrm{score=}\overset{n}{\underset{i=1}{\sum }}\left(Coefi*Expi\right)$ . Here, Coefi is the regression coefficient of the selected TMEM protein family gene and Expi is the expression level of the selected TMEM protein family gene. Patients in each cohort (training, testing, entire, and GSE16091 cohorts) were stratified into high- and low-risk groups according to the median risk score value in the training cohort. Kaplan–Meier survival analysis was performed to compare the overall survival between high- and low-risk groups. The sensitivity and specificity of the TMEM protein family gene-based signature were determined through a time-dependent receiver operating characteristic (ROC) curve analysis.

### Nomogram construction

A prognostic nomogram was developed using the *rms* package to quantitatively analyze the overall survival of osteosarcoma patients. The nomogram integrated clinical factors including gender and age, and the signature-derived the risk score. Calibration curves were plotted to assess the prediction performance of the nomogram by analyzing the consistency of the nomogram-predicted survival with the actual survival.

### Functional enrichment analysis

Osteosarcoma patients of the TCGA entire cohort were stratified into high- and low-risk groups based on the prognostic signature. Differentially expressed genes (DEGs) were screened out according to the following criteria: *P*-value < 0.05 and |log2(fold change)| >1. The *clusterProfiler* package in R was utilized to perform gene ontology (GO) and Kyoto encyclopedia of genes and genomes (KEGG) analyses of these DEGs.

### Gene set enrichment analysis

For gene set enrichment analysis (GSEA), patients of the TCGA and GSE16091 cohorts were classified into high- and low-risk groups based on the prognostic signature. Enriched pathways in the high- or low-risk groups were identified using the GSEA software (version GSEA 4.0.2). Pathways with NOM *P-*value < 0.05 and |NES| > 1 were regarded as significantly enriched.

### Immune cell infiltration and immune microenvironment

The CIBERSORT algorithm was utilized to quantify the proportions of 22 immune cell subtypes infiltrated in each osteosarcoma sample of the TCGA and GSE16091 cohorts according to the gene expression profiles. Next, differences in the abundance of infiltrated immune cell types were compared between high- and low-risk groups in both cohorts. Moreover, single sample GSEA (ssGSEA) was performed using the *gsva* package in R to compare the enrichment score of immune cells and related immune functions between different subgroups in both TCGA and GSE16091 cohorts.

### Statistical analysis

All the statistical analyses and visualization of the results in the present study were conducted using R software (version 4.1.0) and corresponding packages. The Kaplan–Meier method with a log-rank test was utilized to compare the overall survival in different subgroups. In all instances, differences were considered statistically significant when the *P*-value was less than 0.05.

## Results

### Identification of the prognostic transmembrane protein family genes in osteosarcoma

**FIGURE 1:**
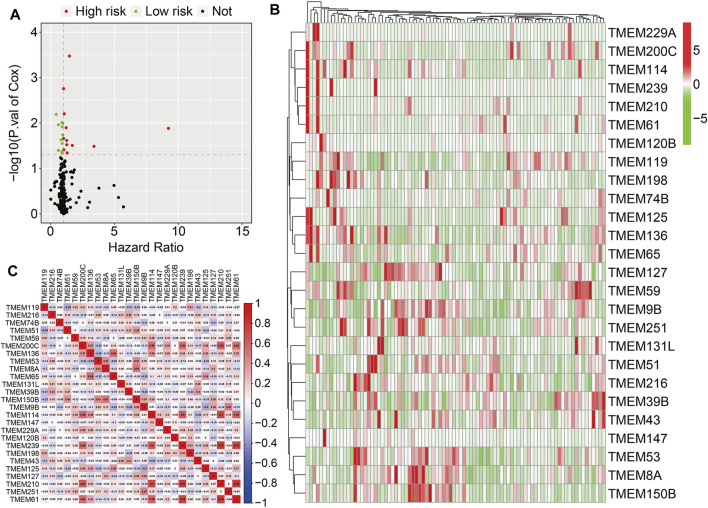
Identification of prognosis-related transmembrane (TMEM) protein family genes in osteosarcoma. **(A)** Prognosis-related TMEM protein family genes were identified by performing a univariate Cox regression analysis. **(B)** Expression profiles of the prognosis-related TMEM protein family genes. **(C)** Correlation of the TMEM protein family genes based on the gene expressions.

A total of 249 well-defined TMEM protein family genes were enrolled in the present study. At first, we conducted a univariate Cox regression analysis to explore the association of TMEM protein family genes with the overall survival of osteosarcoma patients using the dataset from the TCGA database. As shown in Figure 1A, twenty-six prognostic TMEM protein family genes were identified. Of these 26 genes, 15 genes (*TMEM114*, *TMEM239*, *TMEM210*, *TMEM61*, *TMEM125*, *TMEM198*, *TMEM65*, *TMEM59*, *TMEM200C*, *TMEM120B*, *TMEM229A*, *TMEM136*, *TMEM74B*, *TMEM147*, and *TMEM119*) were regarded as risk factors (hazard ratio >1), while the other 11 genes (*TMEM43*, *TMEM127*, *TMEM8A*, *TMEM51*, *TMEM39B*, *TMEM251*, *TMEM9B*, *TMEM216*, *TMEM131L*, *TMEM150B*, and, *TMEM53*) were identified as protective factors (hazard ratio <1). Figure 1B shows the expression profiles of these prognostic TMEM protein family genes and their correlation based on the expression profile is shown in Figure 1C.

### Construction of a prognostic signature based on transmembrane protein family genes

**FIGURE 2:**
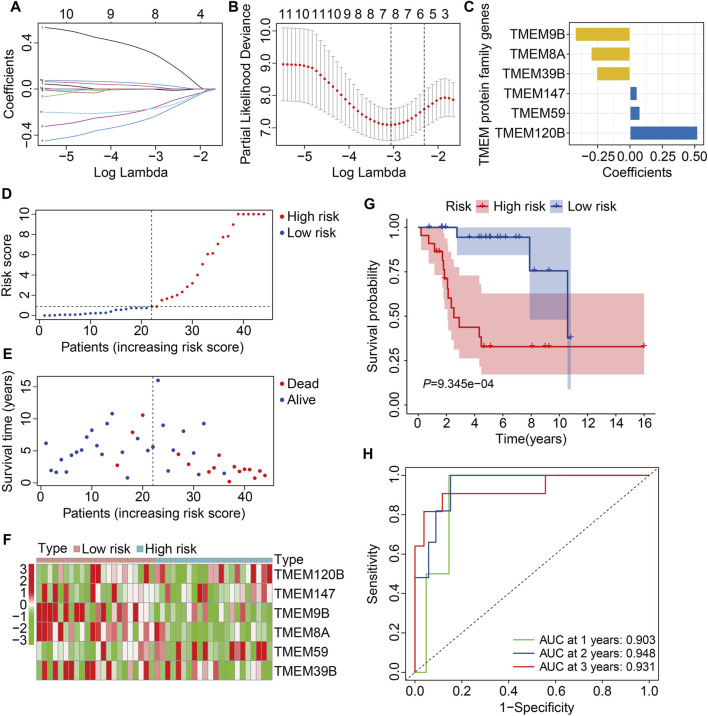
Construction of a TMEM protein family gene-based signature in osteosarcoma. **(A,B)** LASSO regression and multivariate Cox regression analyses. **(C)** The regression coefficients of the six TMEM protein family genes. **(D,E)** The distribution of risk scores and survival status of patients in The Cancer Genome Atlas (TCGA) training cohort. **(F)** Theheatmap shows the expression profiles of the six genes in the high- and low-risk groups. **(G)** Kaplan–Meier survival curve for overall survival in the high- and low-risk groups. **(H)** Time-dependent receiver operating characteristic (ROC) curve for 1-, 2-, and 3-year overall survival.

In the TCGA training cohort, the prognostic TMEM protein family genes were subjected to LASSO-Cox regression analysis (Figures 2A and B), which led to the construction of a prognostic signature based on six TMEM protein family genes. The regression coefficients of the six genes are shown in Figure 2C. The risk score of each individual was determined by a linear combination of the expression levels of the six genes and their regression coefficients. The formula was as follows: risk score = *TMEM120B* × 0.523 + *TMEM147* × 0.054 + *TMEM9B* × (−0.422) + *TMEM8A* × (−0.300) + *TMEM59* × 0.077 + *TMEM39B* × (−0.257). Next, the risk score of each case was calculated and it allowed patients to be assigned to the high- and low-risk groups according to the median risk score value (Figure 2D). Figure 2E shows the survival status and survival time of osteosarcoma patients, and it suggests that the overall survival of patients in the high-risk group seemed to be worse than that in the low-risk group. Figure 2F shows the expression profiles of the six TMEM protein family genes in the high- and low-risk groups. Kaplan–Meier survival analysis demonstrated that patients in the high-risk group had markedly short overall survival compared with patients in the low-risk group (Figure 2G). Then, we performed a time-dependent ROC curve analysis to evaluate the predictive reliability of the prognostic signature. The area under the curve (AUC) values of 1-, 2-, and 3-year overall survival were 0.903, 0.948, and 0.931, respectively (Figure 2H).

### Validation of the transmembrane protein family gene-based prognostic signature in internal and external cohorts

**FIGURE 3:**
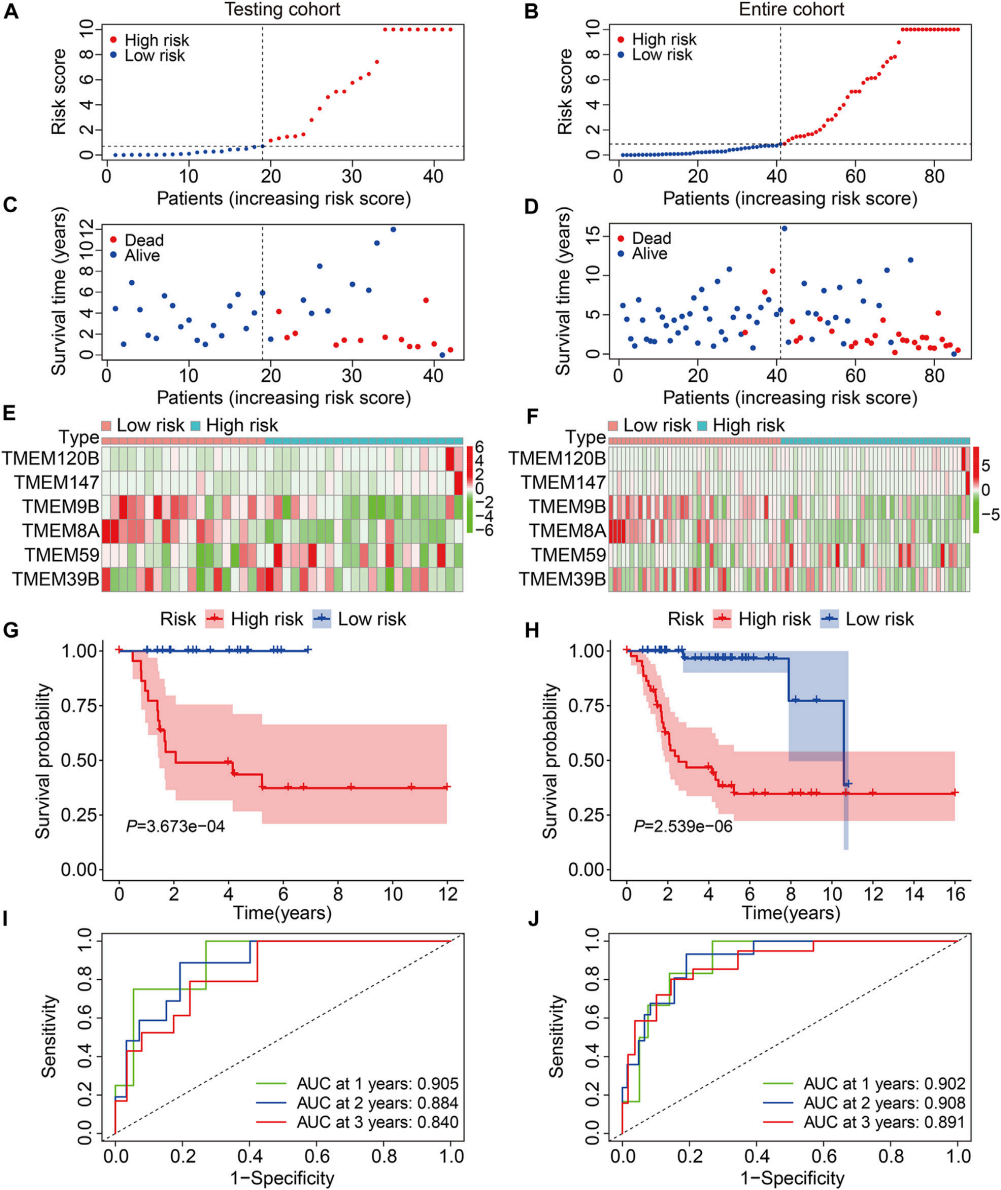
Validation of the TMEM protein family gene-based signature in internal cohorts. **(A,B)** Distribution of risk scores of patients in the testing and entire cohorts. **(C,D)** distribution of survival time and survival status of patients in the testing and entire cohorts. **(E,F)** Expression profiles of the six genes in the testing and entire cohorts. **(G,H)** Kaplan–Meier survival analysis for the comparison of the overall survival between the high- and the low-risk group. **(I,J)** Time-dependent ROC curve for 1-, 2-, and 3-year overall survival in the testing and entire cohorts.

**FIGURE 4:**
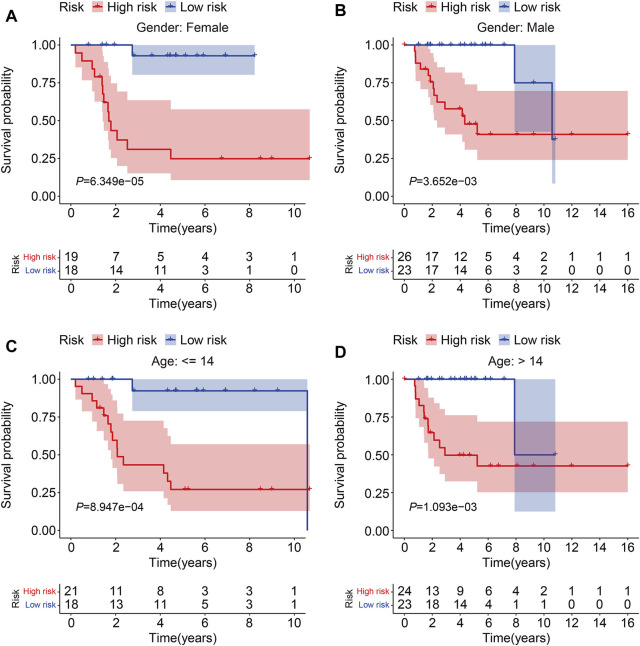
Kaplan–Meier survival curve analysis in different subgroups. **(A,B)** Comparison of the overall survival between high- and low-risk groups in subgroups classified by gender in the TCGA cohort. **(C,D)** Comparison of the overall survival between high- and low-risk groups in subgroups classified by age in the TCGA cohort.

To evaluate the accuracy of the six TMEM protein family gene-based signature, the testing and entire cohorts were utilized for internal validation. Using the same formula as for the training cohort, the risk score of each patient in the testing and entire cohorts was calculated. Then, patients were divided into high- and low-risk groups using the median risk score in the training cohort as the cutoff value (Figures 3A and B). The survival status of each patient in the testing cohort and the entire cohort is described in Figures 3C and D, and it suggests that the mortality rate was markedly increased in the high-risk group than that in the low-risk group. The expression patterns of the six TMEM protein family genes in the testing cohort and the entire cohort are shown in Figures 3E and F. Kaplan–Meier survival analyses indicated that the overall survival of the high-risk group was significantly shorter than that of the low-risk group (Figures 3G and H). The AUC values of the ROC curves at 1, 2, and 3 years were 0.905, 0.884, and 0.840 in the testing cohort (Figure 3I) and 0.902, 0.908, and 0.891 in the entire cohort (Figure 3J). Furthermore, patients in the entire cohort were classified into different subgroups according to their clinical characteristics (gender and age). Kaplan–Meier survival analysis revealed that patients in the high-risk group had worse overall survival compared with those in the low-risk group, which was consistent in all the subgroups (Figures 4A–D).

**FIGURE 5:**
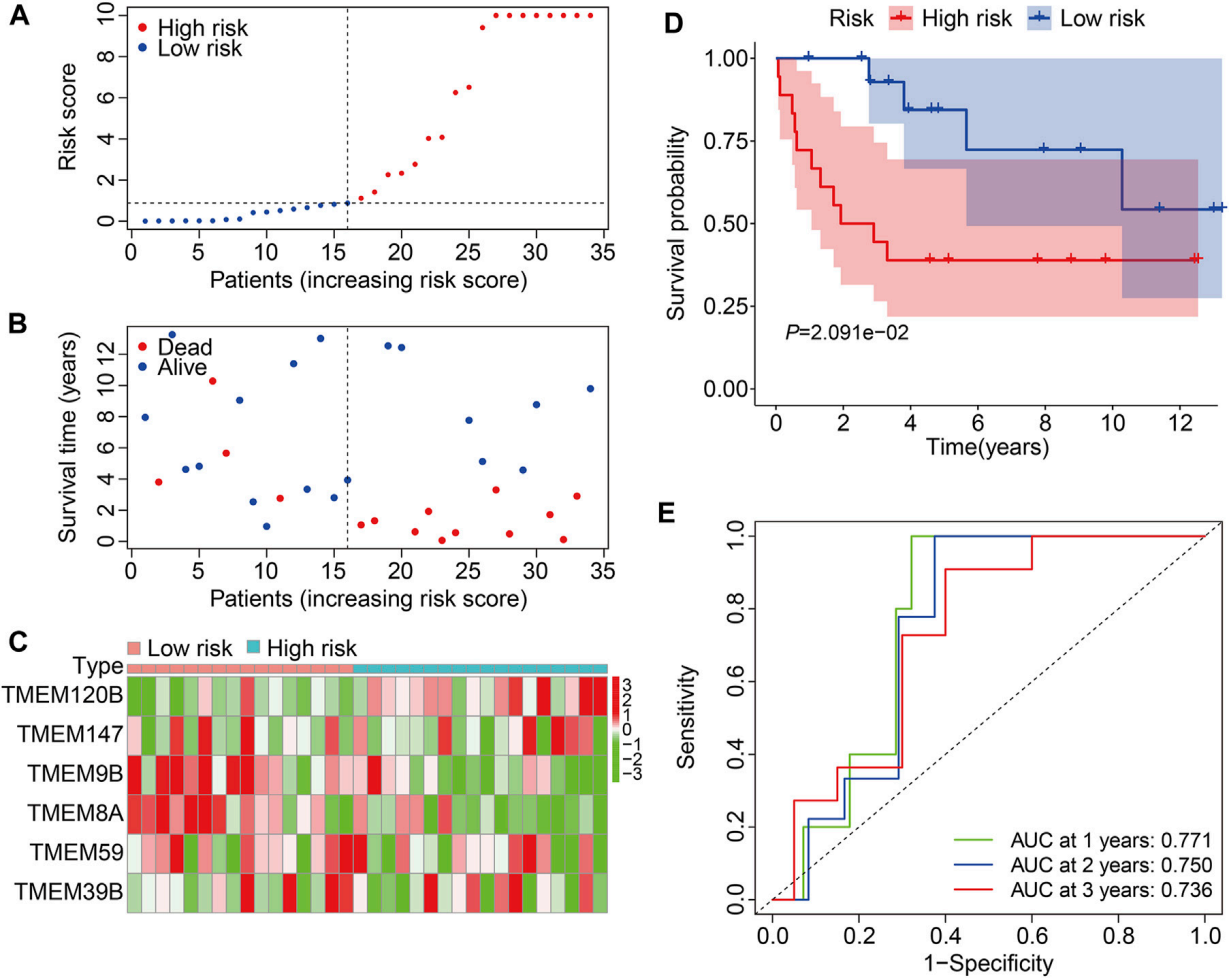
Validation of the TMEM protein family gene-based signature in external cohorts. **(A)** Dot plots exhibit risk score distribution in the GSE16091 cohort. **(B)** Dot plots show the comparison of the survival time and survival status of osteosarcoma patients in the GSE16091 cohort. **(C)** Heatmap for comparison of the gene expression levels in the high- and low-risk groups. **(D)** Kaplan–Meier survival analysis for the comparison of the overall survival between the high- and low-risk group. **(E)** Time-dependent ROC curve for 1-, 2-, and 3-year overall survival in the GSE16091 cohort.

We then employed the GSE16091 cohort for external validation. Using the same formula aforementioned, the risk score of each individual in the GSE16091 cohort was determined and these patients were further stratified into high- and low-risk groups according to the median risk score value obtained from the training cohort (Figure 5A). The survival time and survival status of patients in the GSE16091 cohort are shown in Figure 5B, and it suggests that patients in the high-risk group had a higher mortality rate than those in the low-risk group. Figure 5C shows a comparison of the expression levels of the six TMEM protein family genes between high- and low-risk groups. Kaplan–Meier survival analysis demonstrated that the overall survival was worse in the high-risk group than that in the low-risk group (Figure 5D). The AUC values were 0.771, 0.750, and 0.736 at 1, 2, and 3 years, respectively (Figure 5E). Taken together, these analyses revealed the prognostic robustness of the six TMEM protein family gene-based signature.

### Independent analysis of the prognostic signature and establishment of a nomogram

**FIGURE 6:**
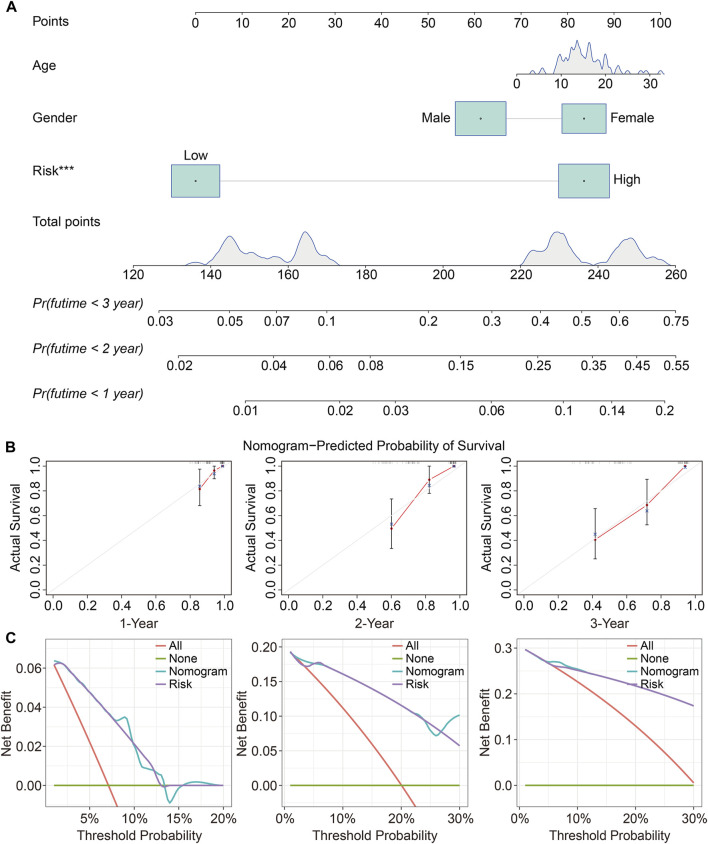
Construction and validation of a prognostic nomogram in osteosarcoma. **(A)** A nomogram was developed by integrating age, gender, and relative risk score. **(B)** Calibration curves exhibit the prediction performance of the nomogram in the TCGA cohort. **(C)** Decision curves for 1, 2, and 3 years.

To determine whether the TMEM protein family gene-based signature could be an independent prognostic factor for osteosarcoma patients, we conducted univariate and multivariate Cox regression analyses in the TCGA dataset. In both univariate and multivariate Cox regression analyses, the risk score, derived from the prognostic signature, was the only indicator of overall survival in patients with osteosarcoma (Table 1). Moreover, we established a nomogram using the relative risk score, gender, and age as variables, and a higher point was related to worse prognosis on the nomogram (Figure 6A). The calibration diagram suggested that the predictive overall survival of the nomogram showed satisfactory consistency with the actual overall survival (Figure 6B). We also plotted decision curves to assess the clinical utility of the nomogram and found that it harbored comparable net benefit for predicting the 1-, 3-, and 5-year survival rates with the signature-derived risk score (Figure 6C), which further indicated that our signature performed well in predicting the prognosis of osteosarcoma patients.

**TABLE 1:** Variables associated with the overall survival in osteosarcoma: univariate and multivariate analyses.

Variables	HR	Univariate analysis		P	HR	Multivariate analysis		P
		95% CI of HR				95% CI of HR		
		Lower	Upper			Lower	Upper	
Gender (female vs. male)	0.68108496462	0.32761977439	1.41589966571	0.30367019218	0.82502920232	0.36115775080	1.88469770668	0.64815565621
Age (≤ 14 vs. > 14)	0.65150759467	0.31318280174	1.35531754477	0.25160818612	0.75222027544	0.33002676463	1.71451349834	0.49817284694
Risk score	1.0000000002	1.0000000001	1.0000000003	0.00175514350	1.0000000002	1.0000000001	1.0000000003	0.00350565086

### Functional analyses based on the prognostic signature

**FIGURE 7:**
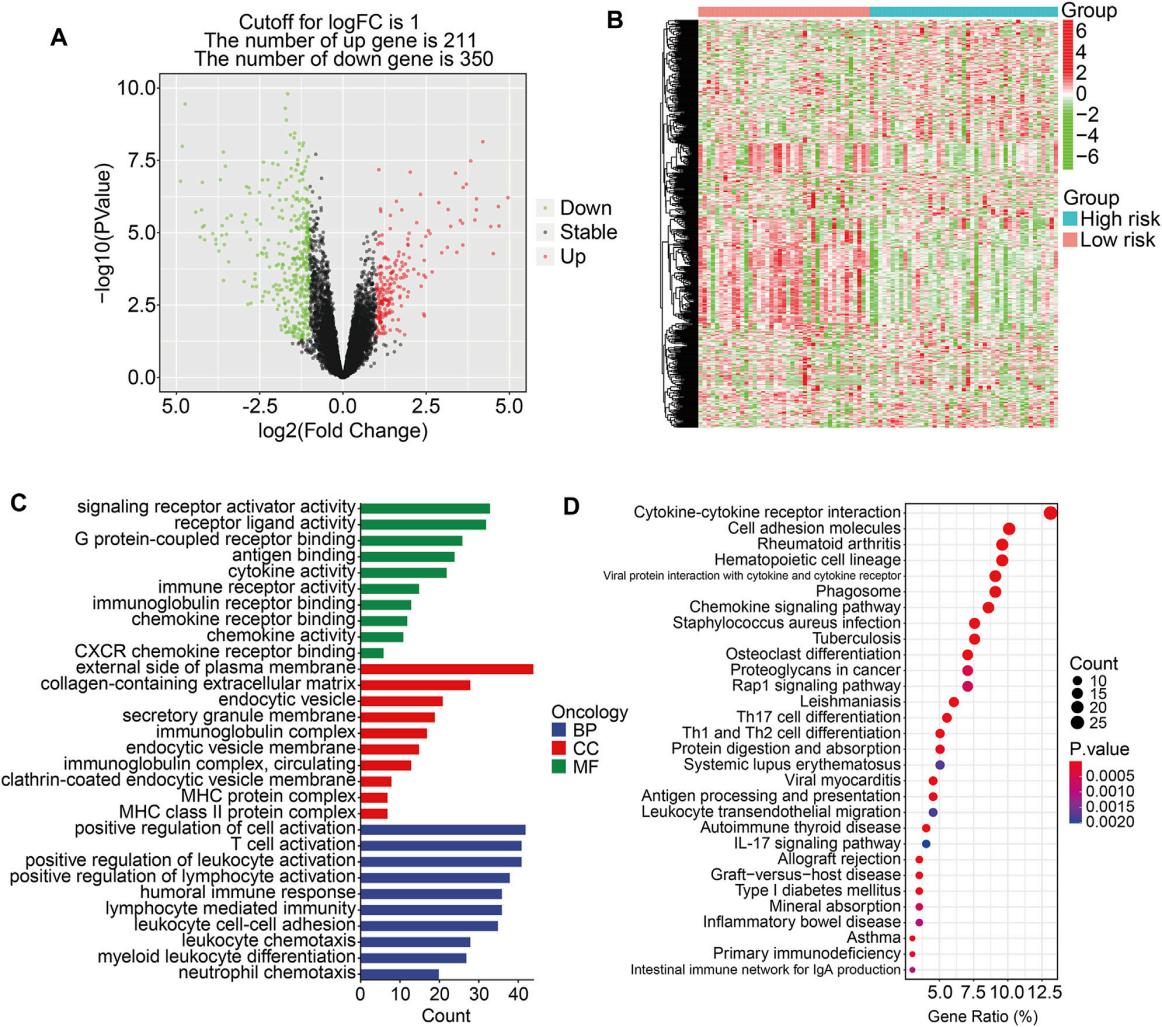
Identification of risk-related differentially expressed genes and functional enrichment analysis. **(A)** The volcano plot exhibits differentially expressed genes (DEGs) between the high- and low-risk groups. **(B)** Heatmap showing the expression profiles of the DEGs. **(C–D)** GO and Kyoto encyclopedia of genes and genomes enrichment analyses of the DEGs.

**FIGURE 8:**
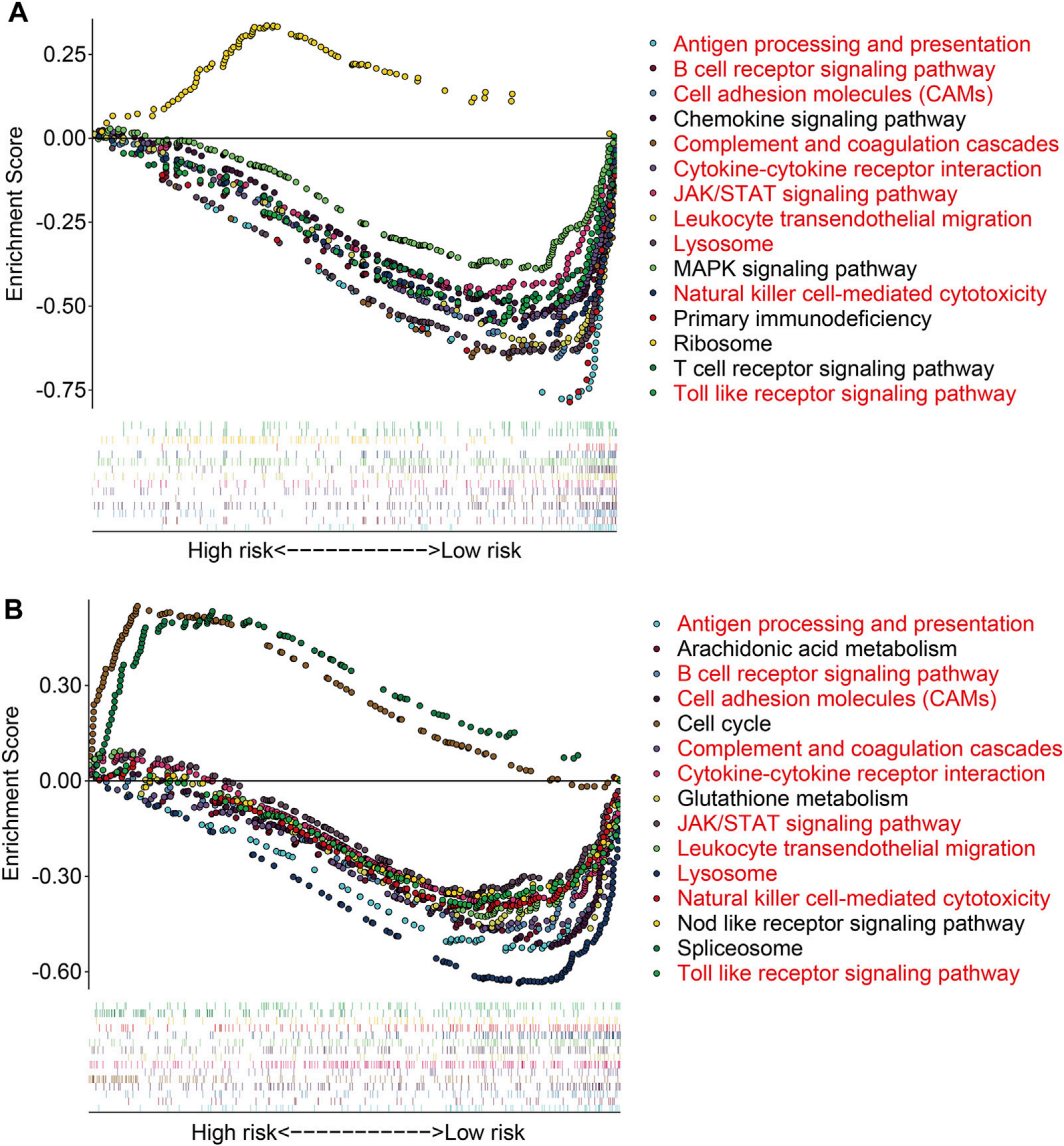
Gene set enrichment analysis between high- and low-risk groups in the TCGA cohort **(A)** and the GSE16091 cohort **(B)**.

To further investigate the potential function of the TMEM protein family gene-based signature, we utilized R package *edgeR* to identify DEGs between groups stratified by the risk score. A total of 561 DEGs were screened out between the high- and low-risk groups in the TCGA cohort with the criteria of *P*-value < 0.05 and |log2FC| > 1. Of the 561 DEGs, 211 were upregulated and 350 were downregulated in the high-risk group compared to those in the low-risk group (Figure 7A). The expression profiles of the DEGs in high- and low-risk groups are shown in Figure 7B. Then, these DEGs were subjected to GO and KEGG enrichment analyses. In the biological process category, DEGs were mainly enriched in positive regulation of cell activation, T-cell activation, positive regulation of leukocyte activation, positive regulation of lymphocyte activation, and humoral immune response. In terms of cellular components, the external side of the plasma membrane, collagen-containing extracellular matrix, and endocytic vesicle were markedly enriched. As for the molecular function, signaling receptor activator activity, receptor ligand activity, and G protein-coupled receptor binding were significantly related to the prognostic signature (Figure 7C). KEGG enrichment analysis revealed that pathways including cytokine–cytokine receptor interaction, cell adhesion molecules, rheumatoid arthritis, hematopoietic cell lineage, and viral protein interaction with cytokine and cytokine receptors were significantly enriched (Figure 7D). Meanwhile, we also performed GSEA to reveal signal pathways associated with the prognostic signature, and the results suggested that immune-related pathways including antigen processing and presentation, B-cell receptor signaling pathway, complement and coagulation cascades, cytokine–cytokine receptor interaction, JAK/STAT signaling pathway, and natural killer cell-mediated cytotoxicity were significantly enriched in both TCGA and GSE16091 cohorts (Figures 8A and B). These analyses illustrated that our TMEM protein family gene-based signature was significantly related to immunity.

### Prognostic signature was associated with the immune microenvironment and immune cell infiltration

**FIGURE 9:**
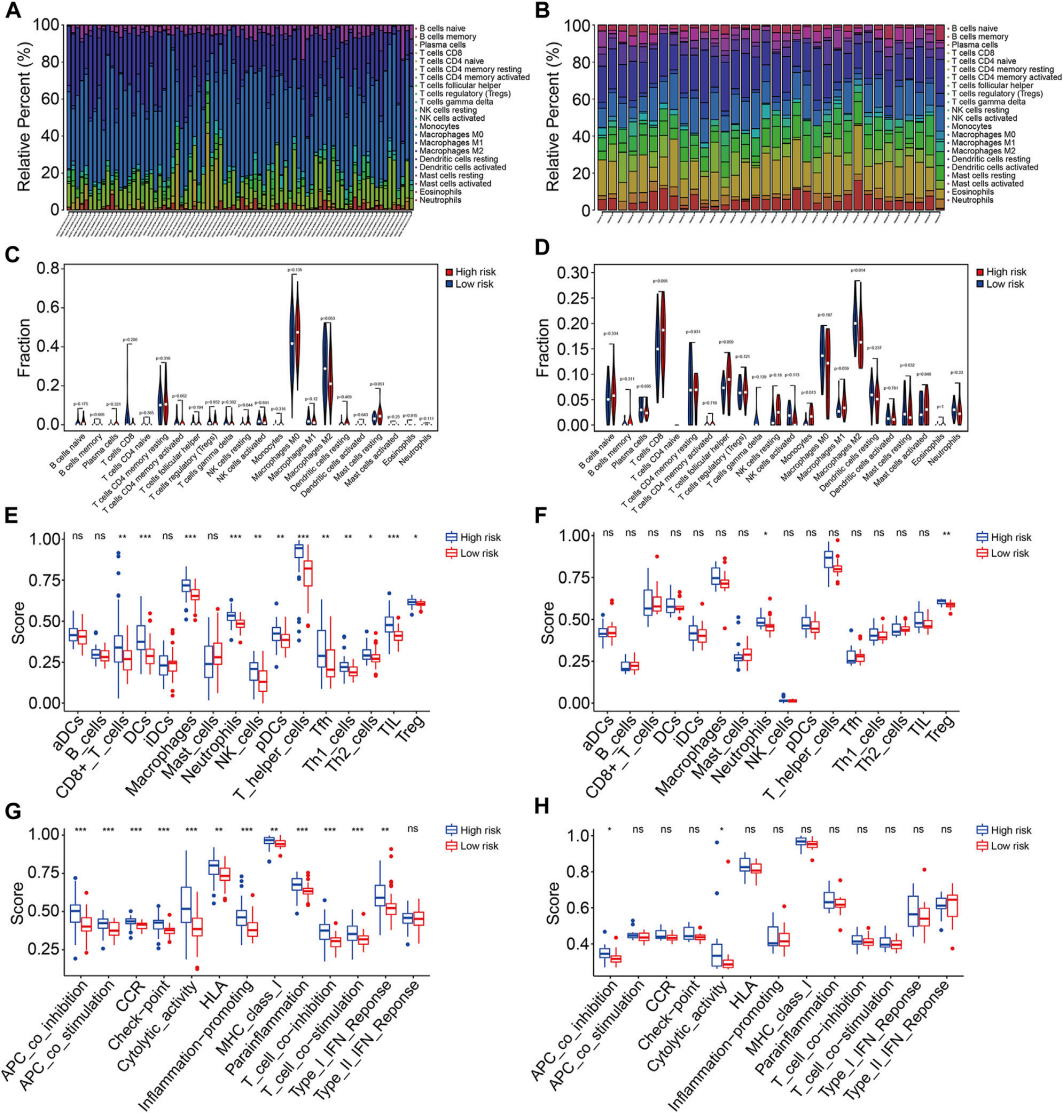
The TMEM protein family gene-based signature was correlated with the tumor immune microenvironment. **(A,B)** Distribution of 22 immune cell types infiltrated in osteosarcoma samples of the TCGA and GSE16091 cohort. **(C,D)** Comparison of the infiltrated immune cells in the high- and low-risk groups of the TCGA and the GSE16091 cohort. **(E,F)** Enrichment scores for the immune cell were compared between the high- and low-risk groups in the TCGA and GSE16091 cohorts. **(G,H)** Enrichment scores for the related-immune function were compared between the high- and low-risk groups in the TCGA and GSE16091 cohorts.

Based on the functional analyses, we further explore the association of the prognostic signature with the tumor immune microenvironment and immune cell infiltration in osteosarcoma. First, we employed the CIBERSORT algorithm to quantify the infiltrated immune cells in high- and low-risk groups. The proportions of 22 immune cell types in osteosarcoma samples of the TCGA and GSE16091 cohorts are shown in Figures 9A and B, and the results revealed that M2 macrophages and M0 macrophages were the most abundant immune cell types in the immune microenvironment. Moreover, we found that high-risk patients with osteosarcoma had a decreased M2 macrophage proportion in both TCGA and GSE16091 cohorts (Figures 9C and D). Furthermore, we also conducted ssGSEA to compare the enrichment scores of immune cells and related immune functions in high- and low-risk groups. In the TCGA cohort, the scores of most immune cell types were significantly different between the high- and low-risk groups, especially the DCs, macrophages, neutrophils, T-helper cells, and TIL (Figure 9E). In the GSE16091 cohort, the scores of neutrophils and Treg were markedly lower in the high-risk group than those in the low-risk group (Figure 9F), which were consistent in both cohorts. As for the related immune functions, APC co-inhibition and cytolytic activity exhibited fewer active levels in the high-risk group than that in the low-risk group in both cohorts (Figures 9G and H).

### Expression and Kaplan–Meier survival analyses of the six transmembrane protein family genes

**FIGURE 10:**
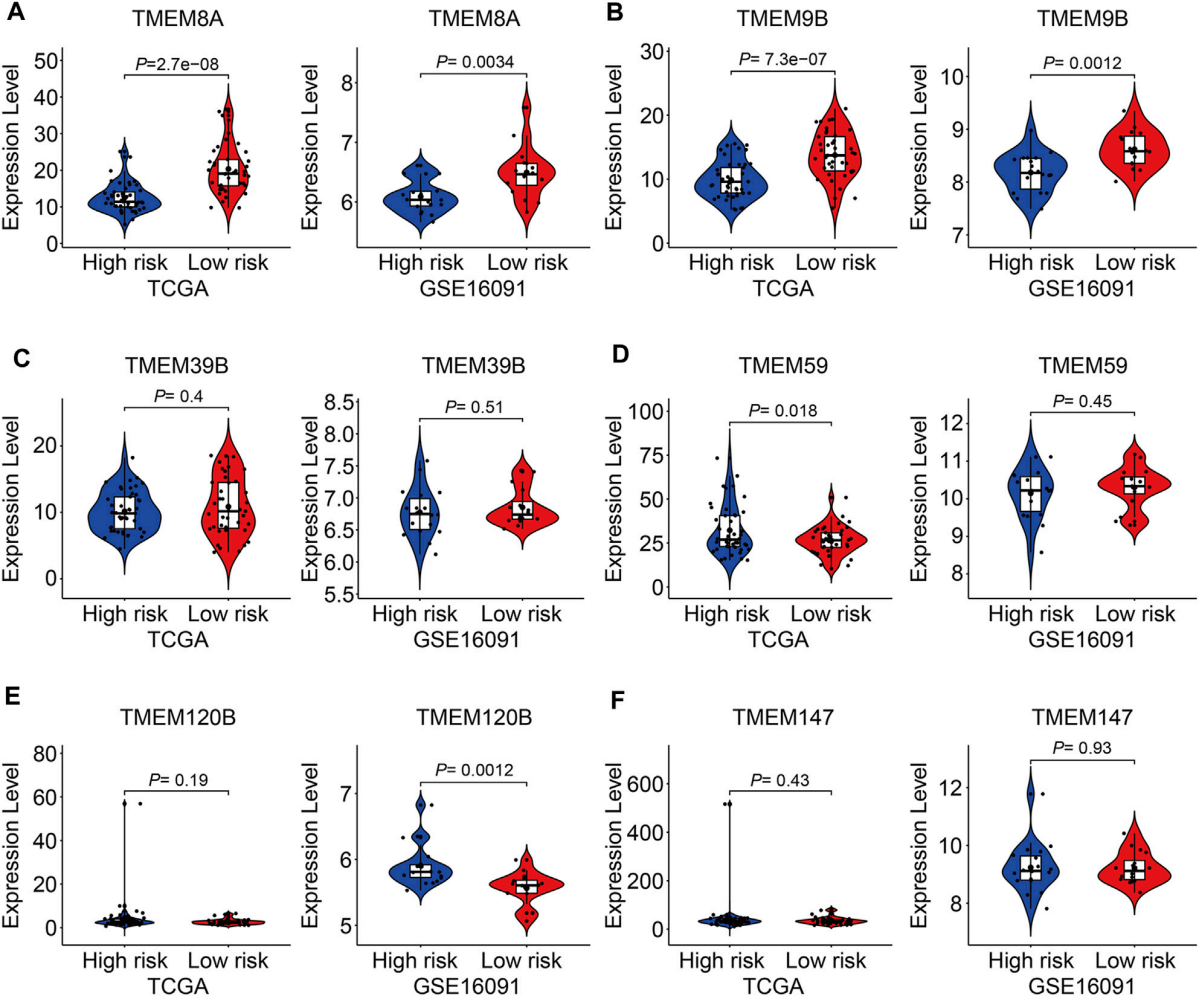
Expression of TMEM8A **(A)**, TMEM9B **(B)**, TMEM39B **(C)**, TMEM59 **(D)**, TMEM120B **(E)**, and TMEM147 **(F)** in high and low-risk groups.

**FIGURE 11:**
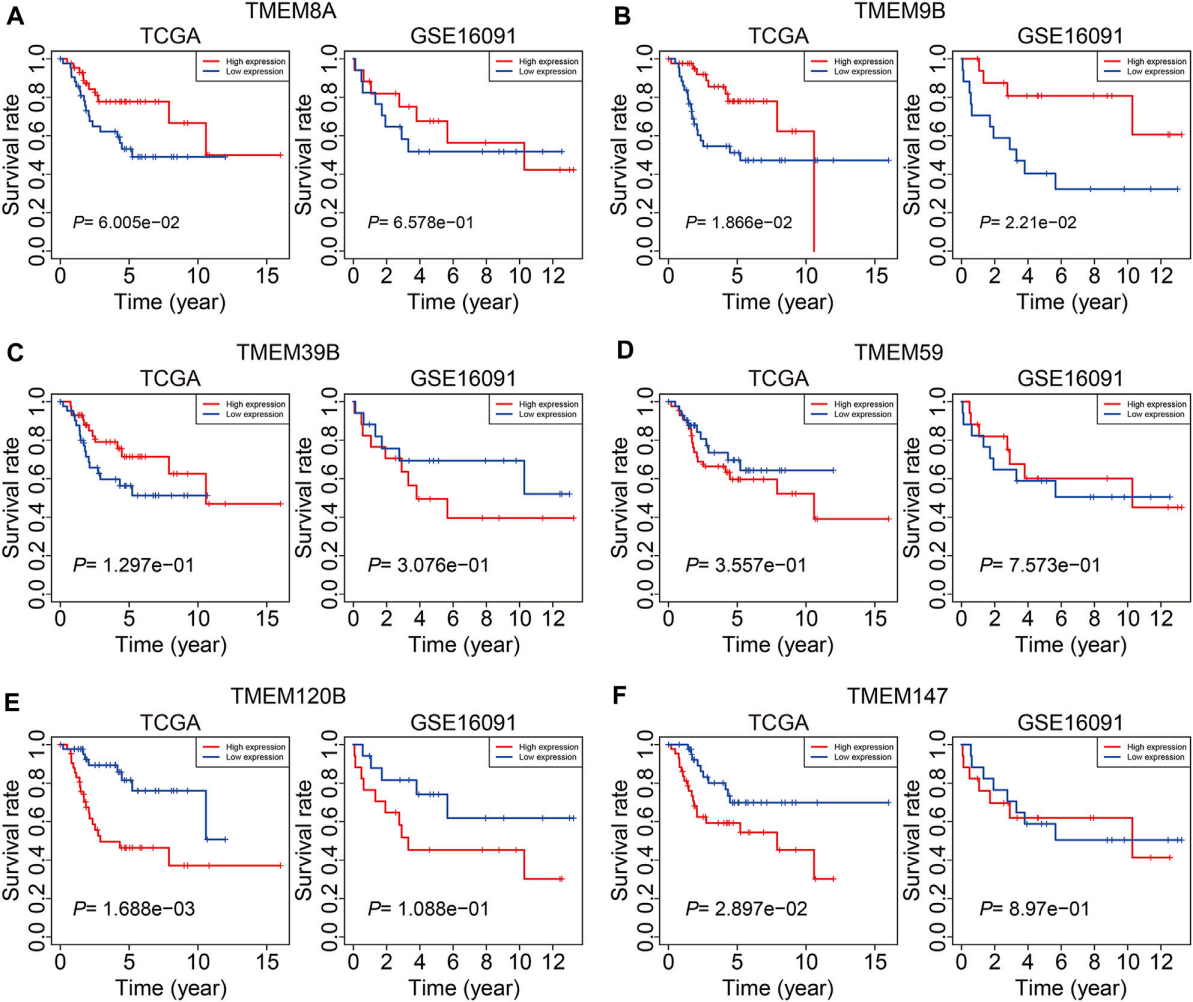
Kaplan–Meier survival curve analysis of TMEM8A **(A)**, TMEM9B **(B)**, TMEM39B **(C)**, TMEM59 **(D)**, TMEM120B **(E)**, and TMEM147 **(F)** in the TCGA and GSE16091 cohorts.

At last, we conducted expression and Kaplan–Meier survival analyses of the six TMEM protein family genes using datasets from the TCGA cohort and the GSE16091 cohort. In both cohorts, the expression levels of *TMEM8A* and *TMEM9B* were lower in the high-risk group than those in the low-risk group (Figures 10A and B), while the expression levels of *TMEM39B* and *TMEM147* exhibited no significant difference between the two groups (Figures 10C and F). Besides, the expression levels of *TMEM59* or *TMEM120B* were higher in the high-risk group in the TCGA cohort or the GSE16091 cohort (Figures 10D and E). Moreover, Kaplan–Meier survival analysis revealed that *TMEM9B* was positively associated with the overall survival of osteosarcoma patients in both TCGA and GSE16091 cohorts (Figure 11B). Higher expression levels of *TMEM120B* and *TMEM147* predicted worse overall survival in patients of the TCGA cohort, while they were not associated with the prognosis of osteosarcoma patients in the GSE16091 cohort (Figures 11E and F). In both cohorts, the expression levels of *TMEM8A*, *TMEM39B*, and *TMEM59* were not related to the overall survival of osteosarcoma patients in the Kaplan–Meier survival analyses (Figures 11A, C, and D).

## Discussion

Osteosarcoma is a kind of malignant tumor with high heterogeneity and it is still a challenge to accurately predict the clinical outcome of patients with osteosarcoma even if they are under standard management (Schiavone et al., 2019; Zhou et al., 2020). In general, the traditional prognostic stratification method is based on the clinicopathological features of tumors, such as tumor size, site, local invasion, and metastasis. However, the accuracy and sensitivity of these features in predicting the prognosis of osteosarcoma patients are limited. With the popularization of high-throughput sequencing technology in cancer research, a growing number of genomic data were uploaded to open-sourced online platforms (Reuter et al., 2015; Hong et al., 2020). Re-analyzing of datasets in the public databases is being a popular and effective tool for the identification of potential therapeutic targets and prognostic biomarkers. In recent times, an expanding list of biomarkers, based on a predetermined gene expression signature, are identified in multiple cancers (Deng et al., 2021; Xu et al., 2021). For example, Wang et al. (2022) developed a TNF family-based signature in diffuse gliomas with regard to prognosis and further explored the association of the signature with the tumor immune microenvironment.

It is known that TMEM proteins participated in oncogenesis and progression. The upregulated or downregulated TMEMs in cancers contributed to cell proliferation, migration, invasion, and metastasis, and these TMEMs were regarded as prognostic markers in multiple types of cancers and therapeutic targets for cancer treatments (Liu et al., 2019; Marx et al., 2020). However, the expression profiles of TMEM genes as well as the corresponding clinical significance in osteosarcoma remain to be elucidated. We here systematically analyzed TMEM protein family genes in osteosarcoma using a dataset from the TCGA database. A total of twenty-six prognosis-related TMEMs were identified by performing the univariate Cox regression analysis and were further utilized to construct a six-gene signature for prognosis prediction in osteosarcoma. Validation in the internal cohort and external GSE16091 cohort demonstrated that the TMEM gene-based signature could discriminate osteosarcoma prognosis with high accuracy. In addition, the signature-derived risk score was the only independent prognostic factor in osteosarcoma as revealed by the univariate and multivariate Cox regression analyses. We also developed a nomogram by integrating the relative risk score and clinical features including gender and age, which could be utilized to conduct personalized survival prediction for each case with osteosarcoma and might be helpful for designing management schedules and decision-making. These analyses suggested that our TMEM protein family gene-based signature is a novel and clinically useful prognostic marker for osteosarcoma patients.

To reveal the potential biological mechanism of our TMEM protein family gene-based signature, we identified risk-related DEGs and performed a functional enrichment analysis. In the GO analysis, immune-related biological processes such as positive regulation of cell activation, T-cell activation, positive regulation of leukocyte activation, positive regulation of lymphocyte activation, and humoral immune response were significantly enriched. KEGG enrichment analysis suggested that risk-related DEGs were mainly enriched in cytokine–cytokine receptor interaction, cell adhesion molecules, rheumatoid arthritis, hematopoietic cell lineage, and viral protein interaction with cytokine and cytokine receptors. Further annotation of the TMEM protein family gene-based signature via GSEA indicated that immune-related pathways including antigen processing and presentation, B-cell receptor signaling pathway, complement and coagulation cascades, cytokine–cytokine receptor interaction, JAK/STAT signaling pathway, and natural killer cell-mediated cytotoxicity were significantly enriched in both cohorts. Thus, our analyses connected our signature with the immunity in osteosarcoma. Then, we explored the association of the TMEM protein family gene-based signature with tumor immune cell infiltration and immune microenvironment. As revealed by the CIBERSORT algorithm, M2 macrophages and M0 macrophages were the most abundant infiltrated immune cell types in osteosarcoma, and patients at high-risk had a decreased M2 macrophage proportion in both cohorts. M2 macrophages are generally considered to promote tumor growth. However, a recent study also suggested that the presence of CD163-positive M2-polarized macrophages is essential for the inhibition of osteosarcoma progression (Gomez-Brouchet et al., 2017), which is in contrast to what is observed in other solid tumors. Thus, the heterogeneous role of infiltrated macrophages in various types of tumors needed to be further explored. A previous study also verified that higher infiltrated M2 macrophages were associated with improved outcomes in patients with osteosarcoma (Zhang et al., 2020). Therefore, infiltrated M2 macrophages in the tumor immune microenvironment were a predictor of prognosis in osteosarcoma. Moreover, we also performed ssGSEA to compare the enrichment scores of immune cells and related immune functions in high- and low-risk groups. In both cohorts, the scores of neutrophils and Treg were markedly lower in the high-risk group than those in the low-risk group. As for the related immune functions, APC co-inhibition and cytolytic activity exhibited fewer active levels in the high-risk group than those in the low-risk group in both cohorts. Taken together, these analyses suggested the immune-suppressive status of the high-risk group, and our TMEM protein family gene-based signature might be utilized to precite the effect of immune therapy.

Of the six TMEM protein family genes comprised in our signature, the expression level of *TMEM9B* was lower in the high-risk group than that in the low-risk group and a lower expression of *TMEM9B* predicted worse overall survival in patients with osteosarcoma, suggesting that *TMEM9B* might act as a protective factor in osteosarcoma. Besides, we found that the expression of *TMEM9B* was decreased in osteosarcoma cell lines compared to that in mesenchymal stem cells by analyzing the GSE70414 dataset in the GEO database (data not shown). *TMEM9B*, mainly located in the lysosome, was a key component of the TNF signaling cascade and was required for the production of proinflammatory cytokines such as IL-6 and IL-8 (Dodeller et al., 2008). In addition, *TMEM9B* was reported to be the downstream effector of the p53-p21 and p16-pRB tumor suppressor pathways, and cells, silencing of *TMEM9B*, would bypass senescence (Rovillain et al., 2011). Despite these findings, the role of *TMEM9B* in malignant tumors had not been reported. Our analyses revealed that *TMEM9B* might exert a tumor-suppressive role in osteosarcoma. Further experiments are needed to explore the effect of *TMEM9B* knockdown or overexpression on the malignant behaviors of osteosarcoma cells and cell senescence. TMEM120B and its paralog TMEM120A were located in the nuclear membrane (Ke et al., 2021). TMEM120B and TMEM120A were reported to be highly expressed in fat and participated in the regulation of adipocyte differentiation (Batrakou et al., 2015). Besides, TMEM120B protein was significantly altered in the aqueous humor of patients with primary open-angle glaucoma (Sharma et al., 2018). However, the exact function of TMEM120B in malignant tumors required further exploration. TMEM147 is a highly conserved membrane protein and is wildly expressed in mammalian tissues and cells (Christodoulou et al., 2020). The cellular sublocalization of TMEM147 varies in different kinds of cells (Maimaris et al., 2021). Though the function of TMEM147 remains largely unknown, it is suggested to be implicated in the regulation of cell proliferation, cell apoptosis, and transcription of target genes (Li et al., 2016). In colon cancer, the TMEM147 expression was significantly increased and might represent a biomarker (Feng et al., 2019). Here, we found that the expression of TMEM147 negatively correlated with the prognosis of osteosarcoma patients in the TCGA cohort. The role of TMEM147 deserved further investigation and it might be a novel therapeutic target in osteosarcoma. TMEM59 is a ubiquitously expressed TMEM protein in human tissues and cells (Liu et al., 2020). In function, TMEM59 could interact with FZD, promoting the formation of multimeric WNT-FZD assemblies and positively regulating the activity of WNT signaling (Gerlach et al., 2018). Besides, TMEM59 mediated autophagy through interacting with ATG16L1 (Boada-Romero et al., 2013). Until now, the role of TMEM59 in tumors is relatively understudied and further experiments need to be performed to investigate the function of TMEM59 in the malignant behaviors of tumor cells.

This study has several limitations. First, not all the TMEM protein family genes were included in our study due to their low expression or lack of them in datasets from the TCGA and GEO public databases. Second, all the osteosarcoma patients used in our study were retrospective cases. Validation of the TMEM protein family gene-based signature in a real-world cohort is necessary. Besides, the effect of knockdown or overexpression of the six TMEM genes, especially *TMEM9B*, on the malignant behaviors of osteosarcoma cells should be further explored and we will conduct it in the future.

In all, we here for the first time investigate the clinical significance of TMEM protein family genes in osteosarcoma. We developed a prognostic signature based on six TMEM protein family genes, which exhibited satisfactory predictive performance in osteosarcoma. Meanwhile, our TMEM protein family gene-based signature was associated with immune cell infiltration and the immune microenvironment. Of the six TMEM protein family genes, lower expression of *TMEM9B* predicted worse overall survival in patients with osteosarcoma. *TMEM9B* might be a potential therapeutic target in osteosarcoma.

## Data Availability

The original contributions presented in the study are included in the article/supplementary material, and further inquiries can be directed to the corresponding author.
